# Tunisian mothers facing the covid-19 pandemic: what are the risks for their mental health?

**DOI:** 10.1192/j.eurpsy.2022.1286

**Published:** 2022-09-01

**Authors:** A. Guermazi, F. Guermazi, M. Bouhamed, M. Ben Abdallah, I. Baati, J. Masmoudi

**Affiliations:** Hedi Chaker University Hospital, Sfax, Tunisia, Department Of Psychiatry A, Sfax, Tunisia

**Keywords:** mental health, Covid-19 pandemic, parental stress, Tunisian mothers

## Abstract

**Introduction:**

Covid-19 pandemic put parents under great pressure, and the most vulnerable parents may have become too overwhelmed to find appropriate ways to be supportive caregivers and to address children’s fears and insecurities.

**Objectives:**

Assess the level of parental stress experienced by mothers during the COVID-19 pandemic and compare it with that experienced by fathers.

**Methods:**

This was a descriptive and comparative analytical study, shared on social networks during the period from 8 to 20 April 2021, targeting mothers of children aged 2 to 18 years. The mother answered the questionnaire for herself and her child. The level of stress experienced by the mother in the parent-child relationship during the COVID-19 pandemic was assessed by the brief version of the Parental Stress Index (PSI-SF).

**Results:**

The total number of participants was 65 mothers. Parental stress level in mothers was high in 58.5%, the average PSI score was 94.25; the mean score of the parental distress subscale was 34.06; the mean score of the dysfunctional child-parent interaction subscale was 27.86; and the average score of the child difficulty subscale was 32.32. The mean scores of the parental distress subscale, the child difficulty subscale, as well as the mean PSI total score were significantly higher in mothers than in fathers, with p= 0.010; p= 0.022 and p=0.017 respectively.

**Conclusions:**

Our results highlight a higher level of stress in mothers than in fathers. This can be explained the parental, marital and professional responsibilities imposed on women, underlining the urgent need to provide mothers with adequate support.

**Disclosure:**

No significant relationships.

